# An evaluation tool for myofascial adhesions in patients after breast cancer (MAP-BC evaluation tool): Development and interrater reliability

**DOI:** 10.1371/journal.pone.0179116

**Published:** 2017-06-09

**Authors:** An De Groef, Marijke Van Kampen, Nele Vervloesem, Sophie De Geyter, Evi Dieltjens, Marie-Rose Christiaens, Patrick Neven, Inge Geraerts, Nele Devoogdt

**Affiliations:** 1 Department of Rehabilitation Sciences, KU Leuven–University of Leuven and Department of Physical Medicine and Rehabilitation, University Hospitals Leuven, Leuven, Belgium; 2 Multidisciplinary Breast Center, University Hospitals Leuven, Leuven, Belgium; 3 Department of Surgical Oncology, KU Leuven–University of Leuven, Leuven, Belgium; 4 Department of Obstetrics and Gynecology, University Hospitals Leuven, Leuven, Belgium; Northwestern University Feinberg School of Medicine, UNITED STATES

## Abstract

**Purpose:**

To develop a tool to evaluate myofascial adhesions objectively in patients with breast cancer and to investigate its interrater reliability.

**Methods:**

1) Development of the evaluation tool. Literature was searched, experts in the field of myofascial therapy were consulted and pilot testing was performed. 2) Thirty patients (63% had a mastectomy, 37% breast-conserving surgery and 97% radiotherapy) with myofascial adhesions were evaluated using the developed tool by 2 independent raters. The Weighted Kappa (WK) and the intra-class correlation coefficient (ICC) were calculated.

**Results:**

1) The evaluation tool for Myofascial Adhesions in Patients with Breast Cancer (MAP-BC evaluation tool) consisted of the assessment of myofascial adhesions at 7 locations: axillary and breast region scars, musculi pectorales region, axilla, frontal chest wall, lateral chest wall and the inframammary fold. At each location the degree of the myofascial adhesion was scored at three levels (skin, superficial and deep) on a 4-points scale (between no adhesions and very stiff adhesions). Additionally, a total score (0–9) was calculated, i.e. the sum of the different levels of each location. 2) Interrater agreement of the different levels separately was moderate for the axillary and mastectomy scar (WK 0.62–0.73) and good for the scar on the breast (WK >0.75). Moderate agreement was reached for almost all levels of the non-scar locations. Interrater reliability of the total scores was the highest for the scars (ICC 0.82–0.99). At non-scar locations good interrater reliability was reached, except for the inframammary fold (ICC = 0.71).

**Conclusions:**

The total scores of all locations of the MAP-BC evaluation tool had good to excellent interrater reliability, except for the inframammary fold which only reached moderate reliability.

## Introduction

Breast cancer is overall the most frequently diagnosed cancer in women worldwide, with an estimated 1.7 million new cases in 2012.[1] Due to the adoption of new treatment approaches survival rate has increased. As a result of different treatment modalities such as axillary and breast surgery, radiotherapy, hormone therapy and chemotherapy, women can have pain and upper limb impairments such as impaired range of motion and lymphedema, leading to limitations in activities of daily living and reduced quality of life.[2]

The occurrence or persistence of upper limb impairments after breast cancer treatment can partially be explained by the presence of myofascial dysfunctions.[3, 4] Myofascial dysfunctions are expressed as myofascial trigger points and adhesions or restrictions of the myofascial tissues. The latter are impairments of gliding of the myofascial tissues relative to each other.[5–9] Muscle manipulation during surgery, scar tissue formation, soft tissue adhesions and adaptive postures following surgery or fibrosis from radiotherapy can cause myofascial adhesions.[3, 4, 7, 9]

Currently, several criteria to determine the presence of myofascial trigger points are established.[6, 10] Most common applied criteria for myofascial trigger points are 1) palpation of a taut band, 2) palpation of a tender point on the taut band, 3) local pressure pain and 4) recognizable referred pain.[10] On the other hand, no method or criteria to evaluate myofascial adhesions exists. Fourie et al determined the presence of myofascial adhesions by palpation for impaired tissue gliding.[7] However, they did not develop a tool and did not investigate his assessments on reliability and validity. Kärki et al, investigated the presence of scar tissue tightness by using a questionnaire.[9]

To our knowledge, only one study investigated the effectiveness of myofascial release techniques in breast cancer survivors, however as part of a multidimensional program.[11] In this study only indirect measurements such as pain and pressure hypersensitivity were used.[11]

However, both in clinical practice and research it is important to verify the presence and the amount of myofascial adhesions in order to be able to direct treatment and to evaluate treatment progress. Therefore, a reliable and valid measurement tool is necessary.

The aim of this study was (1) to develop a tool to evaluate myofascial adhesions in breast cancer survivors and (2) to investigate the interrater reliability of the developed evaluation tool for Myofascial Adhesions in Patients after Breast Cancer (MAP-BC evaluation tool).

## Methods

This observational study was approved by the Ethical Committee of the University Hospitals Leuven (s54579). All participants gave written informed consent prior to their enrollment in the study.

### 1. Development of the MAP-BC evaluation tool

To determine the anatomical locations of myofascial adhesions and how they should be evaluated, two approaches were used. First, literature was searched for studies describing myofascial adhesions and scarring patterns in patients with breast cancer. Secondly, a team of experts was brought together. This team consisted of three manual therapists: one with one year experience and two with more than 5 year experience in treatment of myofascial dysfunctions in patients with breast cancer (ADG, NV and ED). During expert meetings the most relevant anatomical locations for palpation of myofascial adhesions were determined. Consequently, myofascial structures at each location were described and categorised in depth levels. Then, a scoring system and evaluation method was defined. Finally, pilot testing was performed in 15 patients with breast cancer.

For the pilot testing, a convenience sample of 15 women who had surgery for breast cancer were recruited at the Multidisciplinary Breast Centre of the University Hospitals of Leuven between September 2013 and December 2013. Inclusion criteria were unilateral axillary lymph node dissection, breast conserving surgery/mastectomy and presence of myofascial adhesions (determined by clinical examination). Patients with a secondary breast cancer and/or metastasis were excluded. Two therapists of the expert team (ED, ADG) examined the patients independently. Experiences, difficulties and findings were discussed afterwards.

### 2. Reliability of the MAP-BC evaluation tool

The Guidelines for Reporting Reliability and Agreement Studies (GRRAS) were used as a basis to report this reliability study.[12]

The interrater reliability was independently examined by three raters. Two of them (ADG and ED) were members of the expert team. The third rater (SDG) was manual therapist as well with more than 3 years of experience in treatment of myofascial dysfunctions in patients with breast cancer. Prior to the reliability testing they underwent two types of training. First, a 4-hours training session was held for accuracy of the measurements. During this first training, all therapists measured and rated together the same patient at the same moment. Second, training was performed on 20 patients with breast cancer. Inclusion and exclusion criteria for these training-patients were the same as in the pilot and reliability study. During this second training, all therapists evaluated the same patient independently. Results were compared and discussed afterwards.

For the reliability testing, a convenience sample of 30 women who underwent surgery for breast cancer were recruited at the Multidisciplinary Breast Center of the University Hospitals of Leuven. Inclusion criteria were unilateral axillary lymph node dissection and breast conserving surgery/mastectomy. Patients with a secondary breast cancer and/or metastasis were excluded. This cohort was measured between January 2014 and December 2014. Two out of three raters were chosen on the basis of their presence. Measurements took place within a single testing session and within this session the order of the different raters was randomly chosen. Both raters were blinded for the results of each other’s measurements. The possibility of a Hawthorne effect was avoided by making sure the rater was alone in the room during the measurement.[13]

### Statistical analysis

For the total score of each anatomical location the Intraclass Correlation Coefficient (ICC) for single measurements (ICC(2.1)) based on a two-way random effects ANOVA model was used to determine the interrater reliability. The ICC was calculated using SPSS 22. For the different myofascial levels separately, the Weighted Kappa coefficient for agreement between 2 raters was calculated with 95% confidence interval. Additionally, the Absolute Agreement was reported (with 95% Wilson confidence interval) as the proportion of cases in which both raters gave exactly the same rating. Analyses of the Weighted Kappa and Absolute Agreement were performed using SAS software (version 9.4 of the SAS System for Windows). An ICC or Weighted Kappa below 0.50 indicated poor reliability; between 0.51 and 0.75 moderate reliability; between 0.75 and 0.90 good reliability and above 0.90 excellent reliability.[14]

## Results

### 1. Development of the MAP-BC evaluation tool

In the first phase, literature described breast scar tightness in 46% and 29% of patients with breast cancer at 6 months and 12 months after surgery, respectively.[9] Axillary scar tightness was described in 46% and 37% patients at 6 months and 12 months after surgery, respectively.[9] Restricted myofascial tissue gliding was described at the surgical scar (78%), drain sites (29%), axilla and upper arm (83%), axilla and lateral chest wall (61%), posterior axilla/scapula (55%), neck (33%) and other surgical sites (39%).[7]

Following anatomical sites were selected from these two studies describing myofascial adhesions. (S1 File):[7, 9] 1) the scar in the axilla (axillary scar), 2) scar on the breast in case of breast conserving therapy (breast scar) or mastectomy scar in case of mastectomy surgery (mastectomy scar), 3) muscili (mm) pectorales region (the anterior axillary fold), 4) frontal chest wall (the sternum), 5) lateral chest wall (the drain site), 6) axilla and 7) inframammary fold (the place where the breast and the chest meet or used to meet in case of mastectomy). The area ‘axilla and upper arm’, ‘axilla and lateral chest wall’ and posterior ‘axilla/scapula’ described by Fourie et al[7] were reduced to the axilla itself and the lateral chest wall/drain site to avoid confusion on the location for palpation. The locations ‘upper arm’, ‘scapula’ and ‘neck’ were not included because these areas seemed less relevant in the field of myofascial adhesions. According to the experts, these areas should rather be evaluated on the presence of the axillary web syndrome or myofascial trigger points. Additionally, three locations were added by the experts based on information retrieved during their clinical activities (i.e. feedback of patients on self-perceived myofascial restrictions and restrictions frequently palpated by the therapists). First, the mm pectorals region was included because this region is often damaged during breast surgery and by fibrosis after radiation therapy. Second, the frontal chest wall was included because of the possible adhesions of the cervical fascia in this area after radiotherapy. Third, the inframammary fold was added. Especially when a mastectomy was performed, myofascial adhesions could occur in this region.

**Fig 1:**
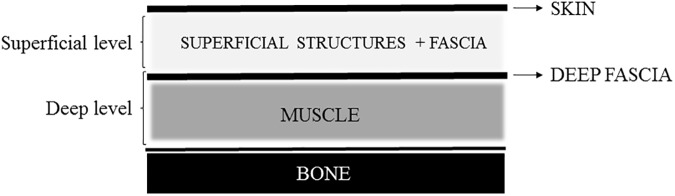
Schematic overview of the different myofascial levels.

During the second phase, myofascial structures for each anatomical location were described. The different myofascial structures were categorised into 3 depth levels: skin, superficial myofascial level and deep myofascial level. A schematic overview is given in Fig 1. The myofascial structures categorised in the superficial and deep level are given in Table 1.

**Table 1:** The different anatomical locations and the description of the myofascial levelsAnatomical location.

	Superficial myofascial level	Deep myofascial level
Axillary scar	The axilla’ with axillary fascia, fat tissue and glands in the axillary pit. *The axilla is bounded by by mm pectoralis (anterior)*, *insertion of m infraspinatus*, *mm teres major and minor and m latissimus dorsi (posterior)*, *m serratus anterior (medial)*, *m subscapulairs (superior)*	mm intercostales externus and internus with the surrounding fascia intercostalis
Breast scar	Subcutaneous fat and glandular tissues of the breast	mm pectorals with the surrounding fascia pectoralis
Mastectomy scar	M pectoralis major and surrounding pectoral fascia	mm intercostales externus and internus with the surrounding fascia intercostalis
Mm Pectoralis region	M pectoralis major and surrounding pectoral fascia	mm intercostales externus and internus with the surrounding fascia intercostalis
Frontal Chest Wall	M pectoralis major and surrounding pectoral fascia	
Lateral Chest Wall	M serratus anterior and axillary fascia	mm intercostales externus and internus with the surrounding fascia intercostalis
Axilla	The axilla’ with axillary fascia, fat tissue and glands in the axillary pit. *The axilla is bounded by by mm pectoralis (anterior)*, *insertion of m infraspinatus*, *mm teres major and minor and m latissimus dorsi (posterior)*, *m serratus anterior (medial)*, *m subscapulairs (superior)*	mm intercostales externus and internus with the surrounding fascia intercostalis
Inframammary fold	Superficial fascia of the abdominal wall	mm intercostales externus and internus with the surrounding fascia intercostalis

During the last phase, a scoring system and instructions for the therapist were developed (S1 File). For the scoring, a 4-points ordinal scale was chosen with 0 no adhesions to 3 very stiff adhesions. Additionally, for each anatomical location a total score between 0 and 9 was calculated. This was the sum of the scores of the 3 levels.

Characteristics of the patients included in the pilot testing are given in Table 2. Both the operated and non-operated side were evaluated. With the exception of one item, both raters agreed on the locations and scoring system of the evaluation tool in its current form. Only for the frontal chest wall, it was decided to include the skin and superficial level only. It was impossible to discriminate between the superficial and deep myofascial level at this anatomical location since the mm intercostales are absent in this region.

**Table 2:** Characteristics of the patients of 1) the pilot study and 2) the reliability study.

	Pilot Study (N = 15) Mean (SD)	Reliability study (N = 30) Mean (SD)
Age (years)	54.6 (17.4)	52.6 (10.0)
BMI (kg/m^2^)	26.5 (4.4)	24.6 (3.8)
Time after Radiotherapy (months)*	7 (3–22)	10 (2–12)
Time after surgery (months)*	9 (4–16)	12 (4–14)
Modified Radical Mastectomy	11 (73%)	19 (63%)
Breast Conserving Surgery	4 (27%)	11 (37%)
Chemotherapy	9 (60%)	16 (53%)
Radiotherapy	12 (80%)	29 (97%)
Endocrine therapy	13 (87%)	25 (83%)
Target therapy	3 (20%)	5 (17%)

* interquartile range is given

### 2. Reliability of the MAP-BC evaluation tool

Thirty women after axillary lymph node dissection for breast cancer were available for reliability analysis. For the lateral chest wall, only 28 measurements were available. Patients characteristics are described in Table 2. In all patients myofascial adhesions were present on at least one anatomical location.

For the total score of each location, the ICC was calculated (Table 3). Interrater reliability of the total scores of the scars were the highest, reaching good (axillary scar, ICC 0.82) to excellent reliability (breast scar, ICC 0.99 and mastectomy scar, ICC 0.96). At all other locations, except for one, good interrater reliability was reached (ICC 0.76–0.87). The ICC for the inframammary fold was the lowest, reaching only moderate reliability (ICC 0.71).

**Table 3:** Interrater reliability (ICC) between two raters for the total score of myofascial adhesions. For each anatomical location the prevalence rate of myofascial adhesions is given.

Location	Rater 1 Median (IQR)	Rater 2 Median (IQR)	ICC (95% CI)	Number (%) of patients with adhesions
Axillary Scar (N = 30)	4 (2.00–6.00)	4 (3.00–6.00)	0.82 (0.65–0.91)	30 (100%)
Breast Scar (N = 11)	0 (0.00–4.25)	0 (0.00–5.00)	0.99 (0.99–0.99)	11 (100%)
Mastectomy Scar (N = 19)	3 (0.00–6.00)	3 (0.00–6.00)	0.96 (0.92–0.98)	19 (100%)
Mm pectorales region (N = 30)	3 (0.75–5.00)	3 (1.00–4.25)	0.87 (0.73–0.93)	23 (77%)
Frontal Chest Wall (N = 28)	2 (0.00–3.25)	1 (0.00–2.25)	0.76 (0.55–0.88)	19 (68%)
Lateral Chest Wall (N = 30)	3 (1.00–4.25)	3 (2.00–4.00)	0.80 (0.62–0.90)	27 (90%)
Axilla (N = 30)	3 (1.00–5.00)	3.5 (2.00–3.50)	0.85 (0.71–0.93)	26 (87%)
Inframammary fold (N = 20)	3 (0.00–3.00)	3 (1.75–3.25)	0.71 (0.48–0.85)	21 (70%)

IQR = Interquartile range; ICC = Intraclass correlation coefficient

**Fig 2:**
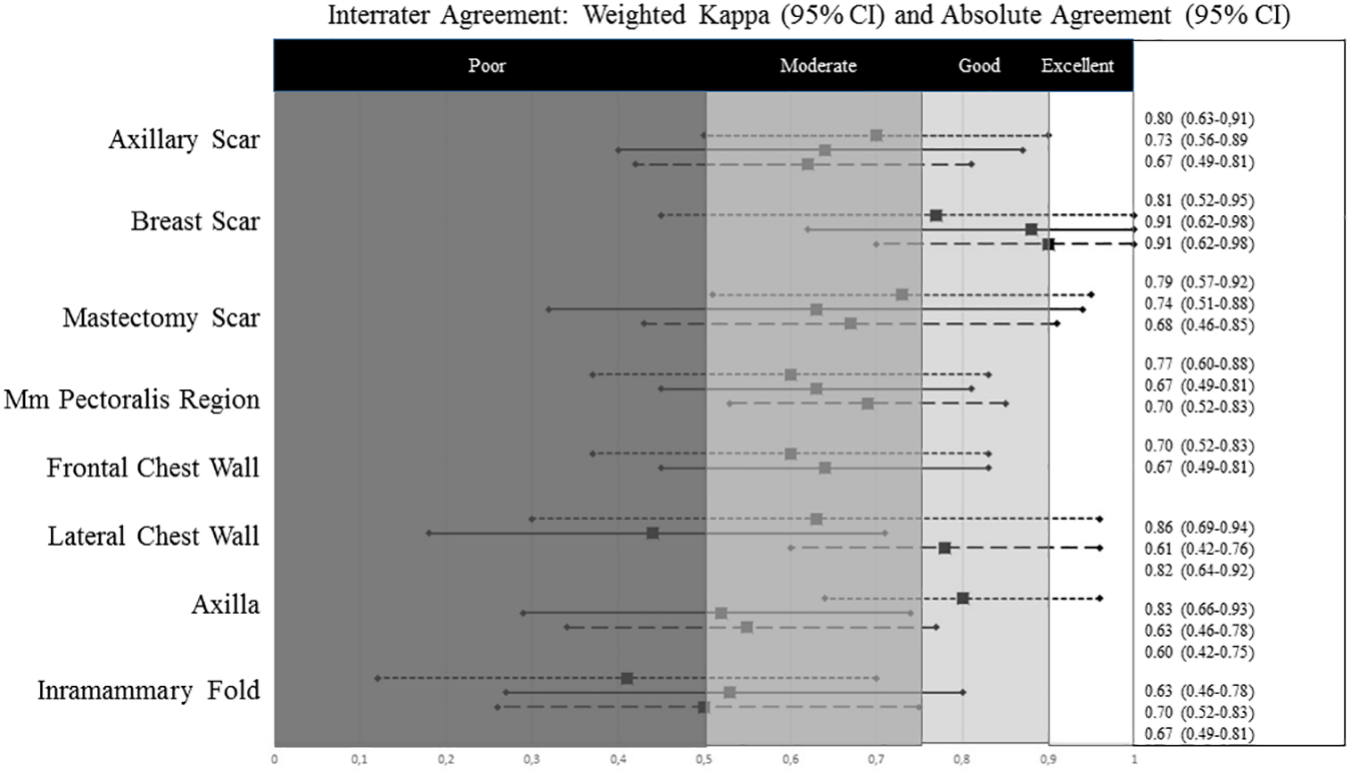
Overview of the Weighted Kappa and Absolute Agreement of the different myofascial levels. The Weighted Kappa (lines), Absolute Agreement (numbers) and their 95% CI for the agreement between two raters on the scoring of the degree of myofascial adhesions for different anatomical locations is given. Per anatomical location the different myofascial levels are given from top to bottom: skin (dotted line)–superficial (full line)–deep (dashed line).

For the different depth levels at each location separately, the Weighted Kappa and Absolute Agreement were calculated. Fig 2 gives an overview of the interrater agreement. For both the axillary and mastectomy scar, moderate agreement was found for all levels (Weighted Kappa 0.62–0.73). The skin had the best agreement. For all levels of the breast scar at least good agreement was found (Weighted Kappa > 0.75). For all scars absolute agreement was higher than 73%, except for the deep level of the axillary and breast scar. For the levels of the other non-scar locations moderate interrater agreement was reached, except for five levels. The deep level of the lateral chest and the skin of the axilla reached good agreement. Interrater agreement of the skin and deep level of the inframammary fold and superficial level of the lateral chest reached only poor agreement. In general, the levels of the inframammary fold scored the lowest (Weighted Kappa 0.41–0.53). Absolute agreement between two raters for non-scar locations ranged between 60% and 86%.

## Discussion

In this study a tool was developed to evaluate myofascial adhesions objectively in patients with breast cancer: the MAP-BC evaluation tool. Additionally, its interrater reliability was investigated. For all 7 locations the total score reached good to excellent interrater reliability, except for the inframammary fold which only reached moderate interrater reliability. Interrater agreement of almost all levels at the different anatomical locations was moderate. Interrater agreement of the inframammary fold was the lowest and interrater agreement of the scars (axilla, breast/mastectomy) was the highest. Absolute agreement between two raters ranged between 60 and 91%, with again the best agreement for the evaluation of the scars.

The interrater reliability of the evaluation of the scars (axilla, breast/mastectomy) was the highest. This may be explained by two reasons. First, these locations were well defined so both raters were more likely to examine exactly the same site. Second, the different myofascial levels were easier to distinguish compared to other locations. At the skin level the linear scar was palpable and the degree of adhesion at this level was determined by the ability of moving the scar itself relatively to the underlying soft tissues. The superficial and deep level of the breast scar reached almost excellent agreement because the superficial myofascial tissues under the breast scar, i.e. fat and glandular tissue, are easy to distinguish from the deep myofascial level. At the mm pectorales region determination of the exact location and distinction of the different levels were expected to be feasible as well. However, interrater agreement values were lower compared to the scars. Possible explanation might be the presence of post-radiotherapy edema or the presence of fat tissue in this region.[15] The same explanations could also be applied for lower interrater agreement values of the superficial level of the lateral chest wall. At this location fat tissue and/or an excess of skin are very common, which made the distinction of the different levels harder. Myofascial adhesions might be present at the lateral chest wall because of the inserted drain after surgery. In the first place, the scar of the drain insertion point was intended to be evaluated. Secondly, the adhesion caused by the drain itself that runs along the chest wall had to be evaluated. The combination of both types of adhesions might have resulted in lower interrater agreement values. Therefore, it was possibly better to score the scar of the insertion point of the drain and the adhesions along the lateral chest wall separately. Interrater agreement of the axilla was moderate, except for the skin level. This may be due to difficult distinction of the superficial and deep myofascial level. The superficial level consists of a package of fat tissue and glandular tissue that should be movable in the axillar pit in all directions. However, after surgery and/or radiotherapy swelling and fibrosis may occur in this region making this package of soft tissues itself harder. Further the presence of the axillary web syndrome may compromise the palpation for adhesions.[16] It may be possible that the scoring of the adhesions was compromised or confused with the hardness of the soft tissues itself. Therefore, it may be useful to score the hardness itself of the soft tissues at the superficial level and the degree of adhesions with the underlying tissues separately. Additionally, the remark should be made that, especially in the axillar region, the starting position of the arm (i.e. 90° abduction) is very important. Lastly, interrater agreement of the inframammary fold was the lowest. Adhesions in this region can be caused by two things. First, during the mastectomy procedure the glandular tissue is removed and the skin is folded towards the thorax. Second, a drain may be inserted in this region after surgery. The inframammary fold region is again a wide region and determination of the exact location for palpation might not have been clear, certainly in case of mastectomy. In general, the inframammary fold is located at the 6^th^ rib, it might have been useful to specify this in the instructions. Likewise at the frontal chest wall, the superficial and deep level might have been difficult to distinguish. Scoring these two levels together at the inframammary fold might have resulted in higher interrater agreement values as well.

In clinical practice, we recommend to use the total scores of the different locations of the MAP-BC evaluation tool. The evaluation takes about 15–20 minutes. The total scores can be used to identify the presence of myofascial adhesions and to direct treatment. Additionally, these scores can be used to evaluate the effect of myofascial therapy or other physical therapy modalities on the myofascial adhesions in patients with breast cancer in a direct way. In line with the evaluation of the frontal chest wall, we recommend to score the superficial and deep level of the inframammary fold together, however interrater agreement was not investigated. The presence of the axillary web syndrome should be registered separately. Before using the MAP-BC evaluation tool, clinical experience in myofascial therapy and a training period is recommended. However, the exact amount of training needed to obtain reliable results should be further explored.

This study has several strengths. First, the measurements were performed in ‘the field’ with the same disadvantages as when performed for clinical purposes, as time-limitations and physical limitations of the patient. Second, the developed tool is a quantitative measurement. Third, the development process of the tool was well established and based on literature, experts and patients experiences.

Some limitations should be mentioned as well. First, in total 30 patients were included but for the mastectomy and breast scar smaller groups of 19 and 11 patients, respectively, were available. Second, only interrater reliability was investigated. Intrarater reliability was not tested since we assumed that intrarater reliability will be as good as or even better than interrater reliability.[17]

The Cosmin Checklist distinguishes three domains in assessing the quality of a measurement instrument, i.e. reliability, validity and responsiveness.[18] The present study focused on interrater reliability of the MAP-BC evaluation tool in breast cancer survivors. Reliability should be further explored in other populations with myofascial adhesions and other scar areas. Further research should explore the validity of the MAP-BC evaluation tool; more specific, content validity, construct validity and criterion validity. In a further stage, responsiveness of the evaluation tool to myofascial therapy should be tested in larger samples. Additionally, the MAP-BC evaluation tool can be used to get more insight in the contribution of myofascial adhesions to pain and upper limb problems in patients with breast cancer. The relationship between myofascial adhesions and different treatment modalities on the one hand and pain and upper limb problems on the other hand should be explored.

In this study the MAP-BC evaluation tool was developed to evaluate myofascial adhesions quantitatively in patients with breast cancer. The total scores of each location had good to excellent interrater reliability, except for the inframammary fold. Almost all myofascial levels at each location reached moderate agreement.

## Supporting information

S1 File The evaluation tool for Myofascial Adhesions in Patients after Breast Cancer (MAP-BC evaluation tool). (PDF)
