# Supplementary material for: An evaluation tool for myofascial adhesions in patients after breast cancer (MAP-BC evaluation tool): Development and interrater reliability
Source: PLoS One. 2017 Jun 9;12(6):e0179116. doi: 10.1371/journal.pone.0179116 (PMC5466317; doi:10.1371/journal.pone.0179116)
Supplement: S1 File — (PDF) [file pone.0179116.s001.pdf]

The evaluation tool for Myofascial Adhesions in Patients after Breast Cancer (MAP-BC evaluation tool)

1. Instructions for the therapists:

Patient is supine, head supported with a pillow and arms next to the body, unless otherwise stated. Therapist stand at the ipsilateral side. Each location is palpated for adhesions at 3 levels of myofascial structures. The degree of adhesions is scored between 0 and 3 at each level. An additional total score can be calculated, i.e. the sum of the scores of the different levels.

Level	Instructions for palpation
Skin	Without any vertical pressure, the skin is moved in all directions relatively to the anatomical structures of the superficial myofascial level.
Superficial	Skin and subcutaneous tissues of the superficial level are moved in all directions relatively to the underlying deep myofascial level.
Deep	All tissues are moved in all directions relatively to the underlying bone structures.

2. Scoring system:

Score	Degree of restricted tissue gliding
0	No restriction in tissue gliding
1	Limited restriction that released immediately
2	Early, hard restriction in tissue gliding
3	Tissue gliding is almost not possible

1. AXILLARY SCAR
(90° abduction)

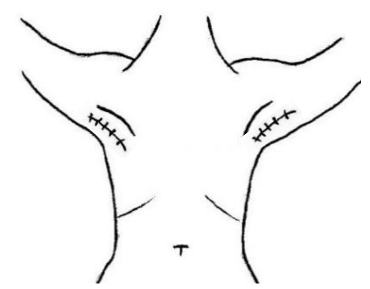

Skin: 0 – 1 – 2 – 3
Superficial: 0 – 1 – 2 – 3
Deep: 0 – 1 – 2 – 3
TOTAL SCORE:

2a. BREAST SCAR
(breast conserving surgery)

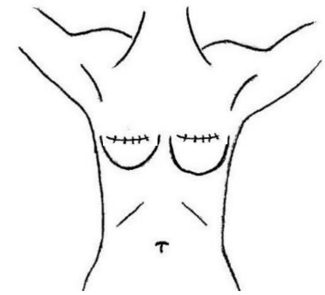

Skin: 0 – 1 – 2 – 3
Superficial: 0 – 1 – 2 – 3
Deep: 0 – 1 – 2 – 3
TOTAL SCORE:

2b. MASTECTOMY SCAR
(mastectomy surgery)

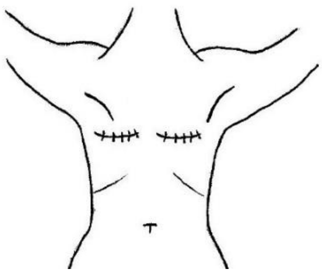

Skin: 0 – 1 – 2 – 3
Superficial: 0 – 1 – 2 – 3
Deep: 0 – 1 – 2 – 3
TOTAL SCORE:

3. MM PECTORALIS REGION
(90° abduction)

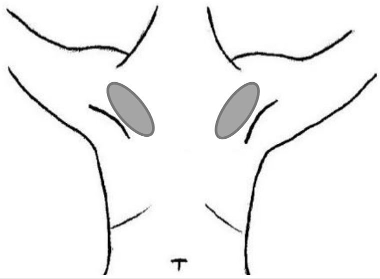

Skin: 0 – 1 – 2 – 3
Superficial: 0 – 1 – 2 – 3
Deep: 0 – 1 – 2 – 3
TOTAL SCORE:

4. FRONTAL CHEST WALL

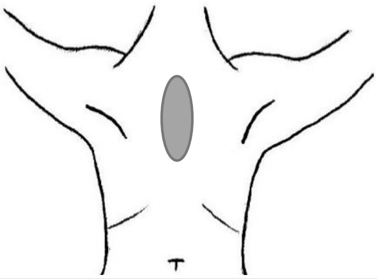

Skin: 0 – 1 – 2 – 3
Superficial: 0 – 1 – 2 – 3
Deep: 0 – 1 – 2 – 3
TOTAL SCORE:

5. LATERAL CHEST WALL

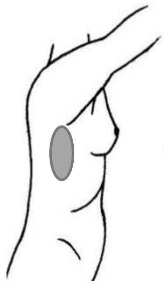

Skin: 0 – 1 – 2 – 3
Superficial: 0 – 1 – 2 – 3
Deep: 0 – 1 – 2 – 3
TOTAL SCORE:

6. AXILLA
(90° abduction)

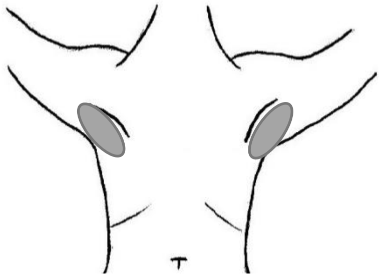

Skin: 0 – 1 – 2 – 3
Superficial: 0 – 1 – 2 – 3
Deep: 0 – 1 – 2 – 3
TOTAL SCORE:

7. INFRAMAMMARY FOLD

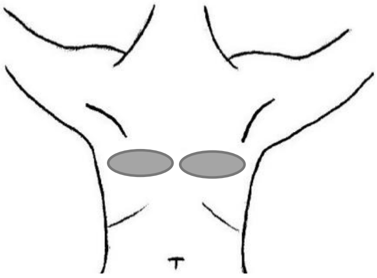

Skin: 0 – 1 – 2 – 3
Superficial: 0 – 1 – 2 – 3
Deep: 0 – 1 – 2 – 3
TOTAL SCORE:
